# Engaging patients and clinicians through simulation: rebalancing the dynamics of care

**DOI:** 10.1186/s41077-016-0019-9

**Published:** 2016-06-15

**Authors:** Roger Kneebone, Sharon-Marie Weldon, Fernando Bello

**Affiliations:** Centre for Engagement and Simulation Science, Imperial College London, Chelsea and Westminster Hospital (Academic Surgery), 3rd Floor, 369 Fulham Road, London, SW10 9NH UK

**Keywords:** Simulation, Sequential simulation, Distributed simulation, Engagement, Co-design, Co-development, Simulation-based re-enactment

## Abstract

This paper proposes simulation-based enactment of care as an innovative and fruitful means of engaging patients and clinicians to create collaborative solutions to healthcare issues. This use of simulation is a radical departure from traditional transmission models of education and training. Instead, we frame simulation as co-development, through which professionals, patients and publics share their equally (though differently) expert perspectives. The paper argues that a process of participatory design can bring about new insights and that simulation offers understandings that cannot easily be expressed in words. Drawing on more than a decade of our group’s research on simulation and engagement, the paper summarises findings from studies relating to clinician-patient collaboration and proposes a novel approach to address the current need. The paper outlines a mechanism whereby pathways of care are jointly created, shaped, tested and refined by professionals, patients, carers and others who are affected and concerned by clinical care.

## Introduction

Patient participation has become a fashionable term, yet there is little agreement about how such participation should be achieved. This paper proposes healthcare simulation-based enactment, co-designed by patients, clinicians and educators together, as a way to bridge gaps in perspective and redress the imbalances of power which suffuse contemporary healthcare. Crucially, such an approach reinforces individual patients’ experience as the essence of every clinical encounter.

Greenhalgh has suggested that, despite its many benefits, the evidence-based medicine movement has had undesirable unintended consequences, displacing attention towards populations and away from individual patients [1]. She argues for ‘real evidence-based medicine’, which requires the involvement of patients. At the same time, a groundswell of opinion is highlighting the need to recapture values and practices which are in danger of being swept away. These include mastery of physical examination, the need for individual clinical judgement and reasserting the primacy of the relationship between wise clinician and patient [2–6]. As sociologist Erving Goffman [7] explains, social experiences are often organised and interpreted in a single primary framework. The reality, however, is much more complex and extends across cultural boundaries.

‘It should be obvious that the human body and touching’s [*sic*] of it will figure in the issue of frame maintenance, just as the body’s various waste products and involuntary movements will figure in tensions regarding boundaries. For it seems that the body is too constantly present as a resource to be managed in accordance with only one primary framework. It seems inevitable that our interpretive competency will allow us to come to distinguish, say, between an arm waved to signal a car and an arm waved to greet a friend, and that both waving’s [*sic*] will be distinguished from what we are seen as doing when we dispel flies or increase circulation. These discernments in turn seem linked to the fact that each kind of event is but one element in a whole idiom of events, each idiom being part of a distinctive framework. And here what is true of Western society is probably also true for all other societies’. p. 36–37

At the heart of any clinical encounter, two people—a patient and a clinician—are held together in a relationship of care. The apparent simplicity of this truism belies the complexity of healthcare and the communication which underpins it. Effective communication, though immediately recognisable, is highly complex and is mediated in a variety of ways. While communication *about* clinical encounters is dominated by speech and writing, communication *within* a clinical encounter takes place as much through touch, gesture, facial expression, tone of voice and silence as through words.

‘Intellectualising’ care by relying on words alone can strip out this communicative richness and lead to serious misunderstanding. Yet many moves to involve patients in the processes of care take place through writing and discussion, thereby missing much of what is important. This privileging of the verbal also places less articulate people at a disadvantage. What is needed is a means of showing, sharing and experiencing, not just talking. Elliott and Williams [8] suggest ‘the development of initiatives which may require professionals to engage in deliberations outside their traditional professional terrains both intellectually and sometimes physically’.

We propose that enactment of care, using the methods and tools of simulation, can simultaneously address multiple perspectives by including diverse participants; that the re-enactment of healthcare practices through physical simulation can provide a means of communicating what words alone cannot convey; and that co-development of simulation-based enactment may allow relationships between patients and professionals to be rebalanced for the benefit of all concerned (through joint and collaborative simulation design efforts, where all participants can evaluate suggested changes) [9–13]. We believe that when front-line staff re-enact their roles in the context of a patient’s journey, deeper understandings can be gained by all and subtleties that might otherwise be missed can be identified. Solutions can be sought in a setting that reduces the power gradients that permeate actual care.

### Simulation at present

To many, the word ‘simulation’ conjures up visions of medical students taking blood from plastic arms, or surgical teams clustered round a high-tech mannikin or virtual reality simulator as they practise their response to an unexpected emergency. Though the value of such activities is unquestioned, such simulation focuses more on technical aspects of care than the subtleties of clinician-patient communication. Teaching resources are often (in Greenhalgh’s words) ‘schematic, fictionalised vignettes in which the sick patient is reduced to narrative “factoids” that can populate a decision tree or a score sheet in an objective structured clinical examination’ [1]. In our experience, this criticism can also apply to simulation, where scenarios are designed by clinicians and patients’ voices are conspicuously absent.

Of course not all simulation requires simulators. For example, simulated (standardised) patient (SP) programmes work with trained actors who stand proxy for actual patients, addressing the subtleties of healthcare consultation with great expertise [14–17]. SPs offer a means of representing patients’ perspectives, drawing on authentic experience while placing it at a ‘safe distance’ and ensuring anonymity. Yet, though widely established across the world, such simulation is usually concealed from public view, restricted to insiders within healthcare education.

Moreover, the primary focus of many simulations is often a task or skill—inserting a urinary catheter, say, or performing a laparoscopic procedure—rather than a holistic consultation. Such simulation views contextual complexity as distracting and filters it out. We argue that simulation offers significant potential to show what happens inside the clinical world, foregrounding rather than concealing complexity. Viewed as a collaborative design process undertaken jointly by patients, clinicians, policymakers and others, simulation can reassert the importance of contextual nuance rather than attempt to eliminate it.

### Engagement through simulation

We therefore propose an alternative framing, one which views simulation as a means of capturing the essence of a clinical encounter through collaboration between all concerned. In the case of patient care pathways, this essence resides in the human relationships between professionals, patients and those who care for them. The process of participatory design in itself can bring insights and widen perspectives.

For more than 12 years, we have been exploring alternative framings of simulation [18, 19]. Our approach is to place a real person (an actor representing the patient) at the centre of the simulation—arguing that clinical practice is always about relationships between people. During our early work on Hybrid Simulation, we worked with SPs linked with models [20]. More recently, we have developed the concept of sequential simulation (SqS), where snapshots from a trajectory of care are concatenated to enact a patient’s care pathway. Distributed simulation (DS) (portable, low-cost yet realistic physical stagings of clinical procedures) presents clinical pathways in a variety of non-clinical venues, including conference centres, sports halls, community centres and public parks [21, 22]^.^ This is an approach acknowledged through the participatory citizenship in healthcare theory; a framework developed to acknowledge how spaces shape levels of participation [23]. This invites participants to respond and share their response with others whose viewpoints may be very different. In this way, the non-verbal is accorded as much (perhaps more) importance than the verbal, opening new kinds of communication. The development of these approaches has widened the scope of our simulations and enabled us to move from ‘traditional’ applications, such as clinical training and education, to wider objectives such as patient participation, co-design and engagement. A clinical commissioning interim manager made the following comment following one of our simulation workshops: ‘You can really feel that this is a real situation. People are talking over each other and you can’t necessarily get your point across, or your point is missed – that is very real and that enables you to keep it in the here and now instead of thinking very theoretically’ [24].

We have tested this approach of co-design, participation and engagement through simulation activities with patients at over 90 engagement settings, ranging from clinical training workshops to public and charity events. The following examples show the breadth of this work and specifically how our simulation tools and methods (SqS and DS) have enabled this reframing.

**Fig. 1:**
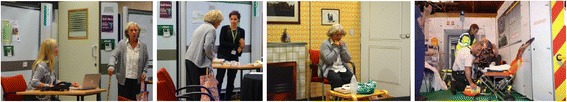
Collaborative scenario development. From *left* to *right*: patient in GP practice; patient with pharmacist; patient at home confused taking new medication; patient attended by the paramedics in their ambulance

(1) A one-day workshop brought 65 elderly diabetic patients, family members, general practitioners (GPs) and other healthcare professionals together to witness a 20-min SqS of an elderly diabetic patient, portraying events unfolding over several days. Small group discussions between the patients and healthcare professionals identified the role of the GP receptionist (not portrayed within the initial scenario) as a crucial but unrecognized element in the pathway. A ‘re-run’ of the scenario, incorporating the receptionist, led to a training programme for GP receptionists across North West London [25] (Fig. 1), followed by workshops for community pharmacists in preparation for a new integrated care approach [26].

Subsequent discussion highlighted differences in perspective between patients, carers and healthcare professionals. In these discussions, all viewpoints were framed as equally valid, escaping traditional hierarchies of perceived importance. Translation from personal experience to a projection onto an enacted scenario provided a safe space in which sensitive issues such as communication, trust and empathy could be explored.

**Fig. 2:**
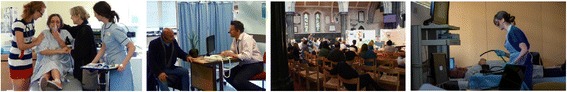
Series of simulation workshops. From *left* to *right*: asthmatic young person in A&E; diabetic patient having GP consultation; participants observing the simulation in action in a church setting; gastroscopy procedure for suspected oesophogeal cancer

(2) A series of SqSs were developed as an educational tool for multidisciplinary teams and young people with asthma [27]. The bespoke SqS tool was used to explore challenges that can arise during an asthmatic patient’s care pathway and the importance of multidisciplinary communication, and to highlight significant issues for all those involved in creating a seamless patient journey. Patients identified that a new role could be beneficial in bridging existing gaps in communication, potentially undertaken by voluntary community members—this role was later termed ‘GP champion’. The same method was later used to recruit GP champions, a role now officially created as an initiative to involve local community members in integrating care. Once recruited and informed about the role through SqS, the GP champions refashioned the role to fit more closely with their own approaches, skills and priorities. The co-redesigned SqS was then showcased to the wider local community in order to raise awareness and promote the GP champion role further.

(3) Two further workshops supported by Clinical Commissioning Groups in London were designed to engage front-line staff, carers, lay members and patients in visualising how a current system works and to co-design a new integrated system [28]. Each workshop started by using SqS to demonstrate the current system, patients and clinicians were then asked to identify areas for improvement and provide potential solutions. Their suggestions were then enacted in a further SqS with the intention of refining and evaluating what worked well and where further improvement was needed. This iterative process allowed new integrated models of care to be designed, tested and refined in collaboration with stakeholders.

(4) A collaboration with the NIHR Diagnostic Evidence Co-operative London (a larger programme designed to support pathway innovation around device technology) explored patient engagement in the development of a volatile organic compound (VOC) breath test for upper gastrointestinal cancer. Through the use of simulation, the VOC research team captured patient attitudes and discussed device usability during the development stage [29]. Figure 2 shows snapshots of these various events.

Several valuable suggestions were made at this early stage by the patients and publics who participated. For example, one unanticipated insight related to the site of the proposed breath testing. Our assumption that a GP practice would be most suitable was challenged by male patients (who constitute a majority of oesophageal cancer sufferers) who pointed out that many men have an aversion to attending their GP surgery. These patients suggested the pharmacy as a more suitable point for testing, as men are likely to buy symptom-relieving medication in the first instance, without consulting a doctor.

**Fig. 3:**
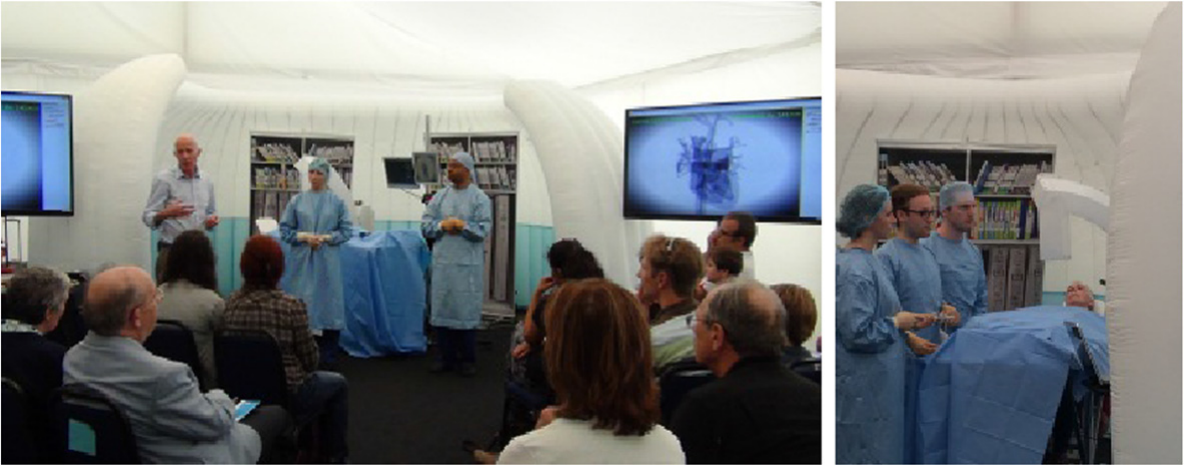
Engaging with coronary angiography. *Left*: audience view. *Right*: close up of team, with audience members as patient (*fourth* from the *left*, reclining) and catheter lab assistant (*third* from the *left*)

(5) A hybrid simulation of elective coronary angiography and stenting under local anaesthetic at the 2014 Cheltenham Science Festival^1^ offered audience members the opportunity to experience cardiological investigation, as an observer, a patient and a member of the clinical team (Fig. 3). This not only highlighted anxieties which clinicians were not aware of or had not thought to explore but also allowed publics to see the clinical team working closely together, commenting on how reassuring they found it. Numerous suggestions for improvement emerged from the discussion, generating an environment in which issues could be shared without evoking defensive responses. Further development has lead to a unique simulation-based training programme for interventional cardiology (‘cath lab’) teams that incorporates clinicians’ interaction with a simulated patient (actor) during procedures under local anaesthesia as one of its key features.^2^

The following quotation from an event attendee highlights the impact of engagement in opening new perspectives: ‘I’m an interventional cardiologist so I do this [angiography] routinely. What’s really interesting is that the first few questions that people asked about how the procedure was going to be. “Will you feel the catheters?” It might surprise you that we often don’t think about that. We do it so routinely. Patients come in. It often feels like a production line at work. As soon as someone gets off the table, someone else is put on. Actually we like to think we do this informed consent process, but often we go through the routine. So actually for me sitting here listening to just a few simple questions from your perspective worrying about the x-rays makes us really focus that we can always do better at explaining things. And actually when you’re giving consent to these procedures, they might feel quite terrifying or frightening. Hopefully it’s less so after you’ve seen it today, but actually as us as the operators [*sic*], I think we can always learn a bit more from just sitting at the back and getting that patients’ perspective. Which is, I assume, most of the rest of you in the room’.

Expressing personal experience through a simulation witnessed by others allows different perspectives to become visible. Detaching the focal activity (a consultation, procedure or care pathway) from its normal clinical setting (hospital or university) loosens the power structures within which professionals carry out their work—Bourdieu’s ‘symbolic capital’ [30]. By ‘taking off their uniform’, clinicians and managers can engage with patients on different terms, gaining fresh insights into what had become familiar.

## Conclusion

We have identified shared experience (which we frame as a process of co-design between patients, clinicians and clinician educators) as a crucial element in creating a participative relationship and propose a way forward. Imaginatively used, healthcare simulation-based enactment can be a valuable resource. Yet this is new territory, and much remains to be learned about co-design and co-development [31]. Establishing a sound scientific footing for engagement through simulation is a priority if we are to restore a missing link in the debate about healthcare—our patients’ genuine participation in the care that affects us all [32].

### Limitations

This field of work covers new ground, and therefore, we only draw on our own experience and research in order to propose simulation-based enactment of care as an innovative and fruitful means of engaging patients and clinicians. In order to understand this approach further, similar approaches and more research is required in the literature.

## Ethics

No ethics were required for this manuscript.

### Consent for publication

Written consent has been obtained from individuals in all images.

## Abbreviations

DS: distributed simulation
GP: general practitioner
ICCESS: Imperial College Centre for Engagement and Simulation Science
NIHR: National Institute for Health Research
SP: standardised patient
SqS: sequential simulation
VOC: volatile organic compound

## Footnotes

1
http://www.cheltenhamfestivals.com/science/

2
http://leadprovider.imperial.nhs.uk/specialty-training/cardiology/teaching-and-training-information/
